# Retraction: Environmental concentrations of metformin exposure affect aggressive behavior in the Siamese fighting fish, *Betta splendens*

**DOI:** 10.1371/journal.pone.0361363

**Published:** 2026-10-26

**Authors:** The PLOS One Editors

Following the publication of this article, the corresponding author made PLOS aware that the last paragraph of the Behavioral assay procedure subsection in the Materials and methods section of [1] is incorrect and that the work reported in [1] was not supported by a valid protocol approval.

A representative of Merrimack College stated that a valid IACUC approved protocol covering the experiments in [1] does not exist, that approval from an external IACUC was not sought, and therefore that the last paragraph of the Behavioral assay procedure subsection in the Materials and methods section of [1] is falsified.

In light of the above concerns about adherence to PLOS’ policy on Animal Research, the *PLOS One* Editors retract this article.

RDM did not respond to the final editorial decision. KW and CM either did not respond directly or could not be reached.
